# Myosteatosis in the Context of Skeletal Muscle Function Deficit: An Interdisciplinary Workshop at the National Institute on Aging

**DOI:** 10.3389/fphys.2020.00963

**Published:** 2020-08-07

**Authors:** Rosaly Correa-de-Araujo, Odessa Addison, Iva Miljkovic, Bret H. Goodpaster, Bryan C. Bergman, Richard V. Clark, Joanne W. Elena, Karyn A. Esser, Luigi Ferrucci, Michael O. Harris-Love, Steve B. Kritchevsky, Amanda Lorbergs, John A. Shepherd, Gerald I. Shulman, Clifford J. Rosen

**Affiliations:** ^1^ Division of Geriatrics and Clinical Gerontology, National Institute on Aging, National Institutes of Health, U.S. Department of Health and Human Services, Bethesda, MD, United States; ^2^ Department of Veterans Affairs and Veterans Affairs Medical Center Baltimore, Geriatric Research, Education and Clinical Center (GRECC), Baltimore, MD, United States; ^3^ Department of Physical Therapy and Rehabilitation Science, University of Maryland School of Medicine, Baltimore, MD, United States; ^4^ Department of Epidemiology, Graduate School of Public Health, University of Pittsburgh, Pittsburgh, PA, United States; ^5^ AdventHealth Translational Research Institute, Orlando, FL, United States; ^6^ Division of Endocrinology, Diabetes, and Metabolism, Department of Medicine, University of Colorado Anschutz Medical Campus, Aurora, CO, United States; ^7^ United States Anti-Doping Agency, Colorado Springs, CO, United States; ^8^ National Cancer Institute, National Institutes of Health, U.S Department of Health and Human Services, Bethesda, MD, United States; ^9^ Department of Physiology and Functional Genomics, University of Florida, Gainesville, FL, United States; ^10^ Intramural Research Program, National Institute on Aging, National Institutes of Health, U.S. Department of Health and Human Services, Bethesda, MD, United States; ^11^ Physical Therapy Program, Department of Physical Medicine and Rehabilitation, University of Colorado Anschutz Medical Campus, Aurora, CO, United States; ^12^ Eastern Colorado VA Geriatric Research, Education, and Clinical Center, Rocky Mountain Regional Veterans Affairs Medical Center, Aurora, CO, United States; ^13^ Sticht Center for Healthy Aging and Alzheimer’s Prevention Wake Forest School of Medicine, Winston-Salem, NC, United States; ^14^ Canadian Frailty Network, Kingston, ON, Canada; ^15^ Department of Epidemiology, University of Hawaii Cancer Center, Honolulu, HI, United States; ^16^ Department of Internal Medicine, Yale School of Medicine, New Haven, CT, United States; ^17^ The Maine Medical Center Research Institute, Scarborough, ME, United States

**Keywords:** myosteatosis, intermuscular adipose tissue, intramuscular adipose tissue, intramyocellular lipids, skeletal muscle function deficit, muscle quality, aging, mobility-disability

## Abstract

Skeletal muscle fat infiltration (known as myosteatosis) is an ectopic fat depot that increases with aging and is recognized to negatively correlate with muscle mass, strength, and mobility and disrupt metabolism (insulin resistance, diabetes). An interdisciplinary workshop convened by the National Institute on Aging Division of Geriatrics and Clinical Gerontology on September 2018, discussed myosteatosis in the context of skeletal muscle function deficit (SMFD). Its purpose was to gain a better understanding of the roles of myosteatosis in aging muscles and metabolic disease, particularly its potential determinants and clinical consequences, and ways of properly assessing it. Special attention was given to functional status and standardization of measures of body composition (including the value of D_3_-creatine dilution method) and imaging approaches [including ways to better use dual-energy X-ray absorptiometry (DXA) through the shape and appearance modeling] to assess lean mass, sarcopenia, and myosteatosis. The workshop convened innovative new areas of scientific relevance to light such as the effect of circadian rhythms and clock disruption in skeletal muscle structure, function, metabolism, and potential contribution to increased myosteatosis. A muscle-bone interaction perspective compared mechanisms associated with myosteatosis and bone marrow adiposity. Potential preventive and therapeutic approaches highlighted ongoing work on physical activity, myostatin treatment, and calorie restriction. Myosteatosis’ impact on cancer survivors raised new possibilities to identify its role and to engage in cross-disciplinary collaboration. A wide range of research opportunities and challenges in planning for the most appropriate study design, interpretation, and translation of findings into clinical practice were discussed and are presented here.

## Introduction

Traditionally the term myosteatosis has been used to describe multiple different adipose depots found in skeletal muscle including: (a) intermuscular adipose tissue (IMAT), the extracellular adipose tissue found beneath the fascia and in-between muscle groups; (b) intramuscular adipose tissue, the extracellular adipose tissue found within an individual muscle; and (c) intramyocellular lipids (IMCL). Myosteatosis is not synonymous with sarcopenia (loss of muscle mass and function); it does appear to be independent of muscle mass and perhaps act synergistically. Thus, many studies have investigated measures of lean or muscle mass and strength, in addition to fatty infiltration. Intermuscular, intramuscular, and intramyocellular fat all provide a slightly different measure of myosteatosis and may represent different risk factors to metabolic and muscle health particularly in older adults. The potential determinants of myosteatosis have not been clearly established, with numerous gaps in the understanding of the pathophysiologic mechanisms that compromise muscle mass, strength, and quality and disrupt metabolism.

The term skeletal muscle function deficit (SMFD) was coined in 2014 to encompass the evolving concepts of sarcopenia and other aging-related muscle dysfunctions that contribute to clinically meaningful mobility impairments (Correa-de-Araujo and Hadley, 2014). In considering other conditions that are clinically manifested as impaired physiologic functions and have multiple contributory factors (e.g., congestive heart failure and chronic obstructive pulmonary disease), this type of diagnostic evolution accommodates both therapeutic progress at a stage when mechanistic information is limited and at further progress as mechanistic understanding increases. In 2017, attention to myosteatosis as a potentially very important component of muscle composition in aging was emphasized in a symposium, in which the need for standardized assessment of myosteatosis was discussed, including imaging methods that can easily and rapidly assess muscle composition in multiple clinical settings and with minimal patient burden (Correa-de-Araujo et al., 2017). Therefore, in building a framework to address muscle quality taking into account myosteatosis in the context of SMFD, it is important to note the complexity of skeletal muscle tissue and its physiologic roles not only in movement *via* force production, but also in metabolism through its maintenance of glucose/insulin homeostasis and amino acid storage (Miljkovic and Zmuda, 2010), thermoregulation, and autocrine, paracrine, and endocrine signaling *via* myokine production (Pedersen, 2011; Ahima and Park, 2015; Colaianni and Grano, 2015). Considering the expanded view of SMFD and its potential multiple components, learning more about the role of myosteatosis in muscle quality and consequently SMFD is critical to allow the development of standardized approaches to its assessment, prevention, and possible treatments. This could, in turn, enhance the quality of life and the potential for healthy and independent living among the fast-growing population of older adults.

The purpose of the interdisciplinary workshop reported here was to gain a better understanding of the roles of myosteatosis in aging, particularly its effects on skeletal muscle function and metabolic disease. Special attention was given to functional status and standardization of measures of body composition (including the value of D_3_-creatine dilution method) and imaging approaches [including ways to better use dual-energy X-ray absorptiometry (DXA) through the shape and appearance modeling] to assess lean mass, sarcopenia, and myosteatosis. The workshop addressed innovative new areas of scientific relevance such as the effect of circadian rhythm and molecular clock disruption in skeletal muscle structure, function, metabolism, and possible contributions to increased myosteatosis. In addition, a muscle-bone interaction perspective compared mechanisms associated with myosteatosis and bone marrow adiposity. Potential preventive and therapeutic approaches highlighted ongoing work on physical activity, myostatin treatment, and calorie restriction. Finally, myosteatosis’ impact on cancer survivors raised new possibilities to identify its role and to engage in cross-disciplinary collaboration.

## Historic Perspectives and Emerging Paradigms

Myosteatosis became an important research focus in humans initially describing the imaging and qualitative characterization of skeletal muscles in Duchenne muscular dystrophy and other neuromuscular diseases (Nordal et al., 1988). Interest in muscle composition led to imaging analysis and development of manual tracings to partition and quantify adipose tissue in the setting of obesity and aging (Goodpaster et al., 2000; Newman et al., 2003). In the Health, Aging, and Body Composition (Health ABC) Study, imaging (computed tomography-CT) quantified muscle, subcutaneous, and intermuscular adipose tissue (SAT and IMAT) in a large aging cohort (70–79 years at baseline) of >3,000 men and women (Newman et al., 2003). Traditional sarcopenia was observed in cross-sectional analyses, with a significant decline in muscle size as people age. The novel finding, however, related to muscle composition defined by attenuation characteristics. Low muscle attenuation (low muscle density) was associated with higher fat content in the muscles, age, and decreased specific force (the functional outcome of reduced muscle quality; Goodpaster et al., 2000, 2001a). This represented the first study demonstrating a correlation between myosteatosis, muscle quality, and weakness in older subjects. Subsequent longitudinal analyses of 5-year data from the same study using serial CT imaging revealed not only classic sarcopenia in men and women who significantly lost muscle over time, but also differing responses between SAT and IMAT (Delmonico et al., 2009). In individuals who lost weight, SAT was decreased in a substantial way, but in contrast, all individuals gained IMAT. This suggests that increases in IMAT are a more consistent signature of aging than traditional sarcopenia. The question remained whether this was a modifiable fat depot and whether physical activity, for example, could affect strength and muscle fat infiltration in the setting of aging.

Investigators from the Lifestyle Interventions and Independence for Elders (LIFE) pilot study attempted to answer the question above by examining the effect of physical activity on mobility-disability in older adults at high risk for disability (Goodpaster et al., 2008). Forty-two individuals were randomized to a physical activity or a control group. Physical activity prevented the age-associated loss of strength, but this finding could not be explained by the prevention of loss of muscle mass. While both groups lost muscle mass, imaging in the control group at “one year of aging,” showed an average 17% increase in IMAT. The physical activity group showed no apparent increase in IMAT but a significant attenuation. IMAT responded uniquely compared to other fat depots as no effect of physical activity was detected in the SAT.

Myosteatosis is also linked to insulin resistance. Using ^1^H magnetic resonance spectroscopy (MRS) to noninvasively assess IMCL content revealed that IMCL was an excellent predictor for muscle insulin resistance in sedentary individuals (Krssak et al., 1999), a finding that has been replicated numerous times (Perseghin et al., 1999; Mayerson et al., 2002; Petersen et al., 2004; Befroy et al., 2007). Consistent with these findings, lipid analysis in muscle biopsies indicate that the IMCL content significantly relates to insulin resistance in patients with type 2 diabetes, obesity, and in lean subjects (Goodpaster et al., 2000; Kelley and Goodpaster, 2001). The higher the IMCL content, the more severe the insulin resistance. Obese subjects with and without diabetes who lose weight essentially reduce their IMCL content and improve their insulin sensitivity. This correlation of findings fits well in the context of an intervention, making the effects of exercise on muscle triglyceride content of great interest. The issue of insulin resistance also points to the complexity of muscle triglycerides. In contrast to those who are obese, an inverse relationship between insulin sensitivity and IMCL content is not observed in highly trained athletes. Despite having a high insulin sensitivity, these athletes show a high amount of IMCL storage (“athlete’s paradox”; Goodpaster et al., 2001b).

Models of insulin resistance disclose two major lipid classes – ceramides and diacylglycerol (DAG), with various studies in mice and rats having demonstrated a strong relationship between muscle DAG content and muscle insulin resistance (Petersen and Shulman, 2017, 2018). These findings were translated to humans where Szendroedi et al. (2014) showed a strong relationship between muscle DAG content, PKCθ translocation and muscle insulin resistance in obese and type 2 diabetic patients, whereas no correlation with muscle ceramide content and muscle insulin resistance was seen in these subjects. In contrast, Amati et al. (2011) found that ceramides were related to insulin resistance, whereas total DAG assessed in a cross-sectional comparison of percutaneous muscle biopsies in insulin-sensitive vs. insulin-resistant subjects was not; analyses of lipid droplet content by electron microscopy recapitulated the athlete’s paradox.

Obese, sedentary subjects and normal-weight trained subjects (men and women, late 60s to early 70s), had about the same amount of muscle triglyceride, and this was consistent across muscle fiber types. Ceramide levels were higher in obese and insulin-resistant individuals compared to insulin-sensitive muscle, but DAG content (total and saturated) was actually higher in insulin-sensitive muscle. These findings made it difficult to distinguish “bad and good” lipids. Some of the ceramides and sphingolipids in the cytosol are related to insulin resistance and some of the DAGs relate to better insulin sensitivity; some of the ceramides or DAGs in the nucleus are linked to either insulin resistance or better insulin sensitivity (Perreault et al., 2018). It is possible that some of these differences relate to muscle-specific differences in myosteatosis. Magnetic resonance imaging (MRI) assessment of the calf identified higher amounts of lipid in the gastrocnemius muscle; lipid in the tibialis and soleus were similar in normal-weight and obese subjects (Perreault et al., 2018). Sphingolipid and ceramide content in muscle biopsies show a robust association with mitochondrial function, with cardiolipin (a marker of mitochondrial content) and some of the genes and proteins related to atrophy signaling (atrogin-1 and caspase-9) being strongly associated with muscles’ ceramide content. Some *in vivo* data indicate that this ceramide content relates to the amount of muscle mass and leg power of an individual. These findings re-emphasize the mechanistic importance of lipid characteristics, as well as the intracellular distribution of lipids in mediating insulin resistance (Cantley et al., 2013).

Studying IMAT vs. IMCL is a very complex area of investigation because the type of muscle, the location of specific lipid species in the muscle, and the current lipidomics technology are very likely to play relevant roles. This opens a wide range of research opportunities and challenges in planning for the most appropriate study design, interpreting and translating findings into clinical practice.

## Myosteatosis and Aging

### Relevant Evidence of Increased Myosteatosis With Aging

With aging, there is a change in body fat distribution resulting in increased ectopic fat accumulations in organs such as the liver, heart, and muscle (Cartwright et al., 2007; Kuk et al., 2009). While multiple cross-sectional studies have examined age-related changes associated with myosteatosis, comparison across studies is not reliable due to non-standardized assessment of myosteatosis including analysis of muscles from different regions of the body. The mid-thigh is the region most commonly investigated, but muscles of the calf, abdomen, and forearm have also been studied. Although increased age affects accumulation of fat in all abdominal muscles, such effect might be larger in the paraspinal compared to the psoas muscle (Anderson et al., 2013).

Fewer longitudinal prospective studies have examined myosteatosis. Song et al. (2004) followed a small sample of 26 African-American older women over 2 years and showed significant increases in IMAT. The Health ABC study also found increased IMAT to be a consistent marker of aging (Delmonico et al., 2009). These findings were replicated in the Tobago Bone Health Study where older African-Caribbean men had almost the same rate of change in myosteatosis as older African-American men (Miljkovic et al., 2016); in addition, it was reported for the first time that myosteatosis accelerates with increasing age among middle aged and older African-Caribbean men. Larger multiethnic population-based studies that recruit individuals without regard to their health status are, however, still needed to further investigate myosteatosis and its relevance to aging. Many of the currently published longitudinal studies focused on community-dwelling populations that were relatively healthy at the start, leaving many questions unsolved about the effects of aging vs. disease as relates to myosteatosis. Few studies investigated longitudinal changes in clinical populations. In an attempt to identify if rates of myosteatosis differ in those with and without osteoarthritis, Beattie et al. (2012) examined rates of change in the quadriceps of women and found that IMAT increased similarly over 2 years in both groups, suggesting that changing rates in myosteatosis are likely more related to aging than disease.

Age-related changes in myosteatosis may depend on several other factors. Evidence shows that women have greater myosteatosis than men, independent of differences in body mass index (BMI) or total body fat (Goodpaster et al., 2001a; Hicks et al., 2005; Ryan et al., 2011; Anderson et al., 2013; Therkelsen et al., 2013; Xiao et al., 2018). Some found no interaction between age and sex, implying that aging was not responsible for the sex difference in myosteatosis rates (Anderson et al., 2013; Xiao et al., 2018). It is possible that sex difference is muscle specific and this may be relevant in population-based studies where only one muscle group has been examined. Further, the role of race and ethnicity in myosteatosis is currently not well understood. Individuals with African ancestry have greater levels of myosteatosis but lower levels of other ectopic fat depots such as fat in the liver or other visceral adipose tissue (VAT). Gallagher et al. (2005) showed that IMAT was greater in African-Americans but only at greater levels of total body fat. Miljkovic et al. (2009) compared African-Caribbean and older white men and found that men of African ancestry had greater IMAT even when matched by age and total body fat. The Health ABC study did not find significant racial differences in the rate of myosteatosis with aging likely because of its much older and relatively healthy population (Delmonico et al., 2009). Little is known about myosteatosis in other racial and ethnic groups particularly in relation to aging. Few aging studies have focused on Hispanics, with most of the research including much younger populations (children and adolescents), making it impossible to extrapolate findings to older adults. In general, Hispanic adolescents have more myosteatosis compared to whites independent of total adiposity. Very limited data are available on older Asian groups and Native Americans. Eastwood et al. (2014) found that differences in myosteatosis between South Asian Indian and white adults disappear when adjusted for total body fat.

Weight history should also be examined in longer longitudinal studies of myosteatosis and aging. Given that myosteatosis is related to both muscle quality and weight gain, it is reasonable to surmise that weight gain with aging may contribute to its increase. Most studies, however, have examined individuals over just a few years, rather than over longer periods of time; an increased length of observation could provide new insights to the role of body weight over a lifetime and changes in myosteatosis.

### Relationship to Mobility, Balance, and Frailty

Myosteatosis is related to increased frailty and decreased muscle and mobility function in both the upper and lower extremities. Shoulder injury in animals and humans shows that fatty infiltration characterizes the disease course and its degree is associated with poorer recovery. Following a tenotomy and neurotomy in the rotator cuff injury in a mouse model, a large and accelerated increase in the accumulation of adipose tissue was observed (Kim et al., 2012), with this increase generally related to a negative prognostic factor for recovery from a rotator cuff tear. As previously mentioned, fatty infiltration is inversely related to muscle specific force, suggesting that as IMAT increases, even when normalized for muscle cross-sectional area, muscle quality decreases (Goodpaster et al., 2001a; Hilton et al., 2008). The decreased muscle quality may partially explain why recovery from a rotator cuff tear and general mobility is worse in those with greater myosteatosis. Multiple studies have found a similar relationship between increased myosteatosis in the lower extremities and decreased mobility (Tuttle et al., 2011, 2012; Marcus et al., 2012).

Numerous studies that examined the relationship between myosteatosis and physical function were done cross-sectionally. However, Beavers et al. (2013) used longitudinal data from the Health ABC study to investigate which changes in body composition measures were most influential in predicting changes in gait speed. Only thigh muscle area and intramuscular fat were significant predictors. Those who maintained their thigh muscle area also maintained their gait speed, whereas those with greatest accumulation of thigh IMAT had greater declines in gait speed. It is unknown whether the increases in myosteatosis were casually related to the declines in gait speed, or if it was simply markers of dysfunction. Using P16-expressing cells (associated with cellular senescence), Justice et al. (2018) showed a very strong link between the abundance of senescent-like cells and IMAT, and that this was strongly and inversely associated with functional measures like gait speed and Short Physical Performance Battery (SPPB) score. Whether this represents the senescent burden of the whole body, or if there is some connection between the secretome of the senescent cells in the intramuscular fat and the muscle is unknown, but this study provides one possible causal pathway for the inverse relationship between myosteatosis and mobility.

Research that examines the relationship between myosteatosis and frailty has relied on performance-based measures of function as a proxy measure of frailty. Studies that show decreased SPPB score (or other similar tests such as the modified physical performance test) are related to increased myosteatosis in lower extremity muscles (Hilton et al., 2008; Tuttle et al., 2011, 2012; Anderson et al., 2016). The majority of studies examining myosteatosis and mobility have been conducted in community-dwelling individuals (Visser et al., 2002; Addison et al., 2014a,b, 2017; Inacio et al., 2014; Anderson et al., 2016). Consistent with studies of aging and mortality, the muscles examined across studies looking into myosteatosis and mobility most frequently used thigh muscles because of the ease to assess the muscle with imaging, biopsies, and strength tests. Several other lower extremity muscles are also important for mobility. Several smaller studies have examined the calf muscles (Hilton et al., 2008; Tuttle et al., 2011, 2012) and few have also studied core muscles including the abdominal and trunk muscles (Anderson et al., 2016; Lorbergs et al., 2019).

More proximal muscles of the lower extremities including the hip abductors have also been the focus of investigations about myosteatosis and mobility (Addison et al., 2014a,b, 2017; Inacio et al., 2014). While balance and falls risk have not been as well researched in the context of myosteatosis and general mobility/function, several studies examined the relationship of myosteatosis and the risk of falls in community-dwelling older adults. These studies generally report that increases in myosteatosis in the abdominal, psoas and hip muscles, relate to increased gait variability (Addison et al., 2014a,b), kyphosis progression (Lorbergs et al., 2019), decreased balance (Addison et al., 2014a,b), and an increased risk of falls (Visser et al., 2002; Addison et al., 2014a,b, 2017; Inacio et al., 2014). Inacio et al. (2014) studied the psoas, multiple muscles in the thigh, and the hip abductors to see if changes in fatty infiltration were occurring equally between the muscles in community-dwelling adult fallers and non-fallers (self-reported fall in the last year). While lean tissue was similar between both groups, fallers had increased amounts of myosteatosis across all muscles, with significantly higher amounts noted in the gluteus maximus and medius/minimus muscles, suggesting more proximal muscles may be vulnerable to myosteatosis and relevant to balance function in older adults.

The relationship of impaired gait and stepping as it relates to increased myosteatosis is also an area of interest. Among individuals divided into impaired steppers and non-impaired steppers, those with impaired stepping response (meaning people who cannot recover as quickly from a balance perturbation) are more likely to suffer a fall (Addison et al., 2017). These individuals showed higher amounts of myosteatosis in their hip abductors and a lower hip abductor torque production, suggesting fatty infiltration of the hip abductors may be important to evaluate when looking at balance and mobility changes in older adults. Small sample sizes have been a concern in many studies examining the relationship of balance, mobility, and myosteatosis, which limits any conclusive interpretation. Difficulty with extrapolating results emphasizes the need for further investigations in larger studies.

### Myosteatosis in Cancer Survivors and Its Application to Aging

Recent studies show low/normal weight (BMI) is associated with poorer outcomes including increased mortality for cancer survivors when compared to obese cancer survivors – a phenomenon called “the obesity paradox” (Strulov Shachar and Williams, 2017). However, the biological basis for this paradox is not fully understood (Cespedes Feliciano et al., 2018; Lee and Giovannucci, 2019). In 2017, the NIH National Cancer Institute (NCI) hosted a workshop, “Understanding the Role of Muscle and Body Composition in Studies of Cancer Risk and Prognosis in Cancer Survivors.” The focus was on body composition and muscle and their effect on cancer progression, survival, long-term effects of treatment, cancer recurrence, and the risk of subsequent cancers. Discussions involved determining potentially meaningful cutpoints for muscle and adipose tissue to identify survivors at risk of poor outcomes and strategies to implement viable interventions.

Given the large variability in types and treatments of cancer, it is currently unknown whether myosteatosis can provide information about cancer survival or risks for poor outcomes including cancer outcomes measures of weight or body composition that are more likely to be routinely collected. Sjoblom et al. (2016) showed that skeletal muscle radiodensity is prognostic for survival in patients with non-small cell lung cancer. Those with a higher radiodensity (indicating a lower level of myosteatosis) had increased rates of survival. Patients with higher levels of myosteatosis in colorectal cancer also demonstrated longer hospitalizations compared to those with lower levels (Martin et al., 2018). Health providers are a potential resource to further examine CT scans as to the relationship of myosteatosis and cancer survival. Kaiser Permanente Northern California, a large health provider in the USA, conducted two studies using CT scans to investigate colorectal and breast cancer in about 3,000 stage 1 through 3 survivors in each group. In the cohort of colorectal cancer, a 30% increase in mortality for those with low muscle and a 20% increase in mortality for those with high adiposity were observed. For individuals with low radiodensity (indicating high levels of myosteatosis), rates were even higher at 60% increase in mortality. Similar association was not found for breast cancer and radiodensity. These studies illustrate the importance of not only examining different cancer sites and types of populations, but also looking at more than just body composition data as sarcopenia and myosteatosis may involve examination of different parameters. In a group of 100 participants with pancreatic and periampullary adenocarcinomas, 40% were sarcopenic, 25% had myosteatosis, and 11% had both (Stretch et al., 2018). The trajectories for survival between these groups differed as those with high levels of myosteatosis had overall lower survival rate. This is clear evidence that further investigations of myosteatosis in cancer survivors are warranted. Body composition alone is unlikely to provide all the information needed. The identification of common data and reliable methodologic resources that allow for a comparison between different centers will also allow clinicians, oncologists, and researchers to expand the field with additional studies that may identify the impact of myosteatosis in cancer survivors and help sharing and applying relevant findings.

## Myosteatosis Potential Determinants and Clinical Consequences

The biological mechanisms of increased myosteatosis are currently unknown, though multiple theories have been presented including defective leptin signaling (Koteish and Diehl, 2001), the involvement of skeletal muscle precursor stem cells (Kirkland et al., 2002) or fibroadipogenic precursor (FAP) cells (Farup et al., 2015), neuromuscular changes resulting in decreased regenerative capacity (Takano et al., 2018) and mitochondrial dysfunction (Schrauwen-Hinderling et al., 2007; Bournat and Brown, 2010).

Myosteatosis has been implicated in numerous negative aging-related outcomes, with resultants such as mobility-disability, metabolic impairments, and increased mortality making myosteatosis a priority research area to help clinicians identify and manage at risk older adults. Myosteatosis has been reported as an independent risk factor for hip fracture, diabetes, disability, hospitalization, mortality, severe illness outcome, and surgery in the very ill. Two studies [Health ABC; Age, Gene/Environment Susceptibility Study in Reykjavik (AGES)] looked at the attenuation of muscle as it would predict hip fractures. In both studies, even when controlling for body composition, the muscle attenuation was associated with increased risk of fracture (Lang et al., 2010; Johannesdottir et al., 2012; Frank-Wilson et al., 2018).

Low skeletal-derived muscle density has been linked to higher 6-month mortality in mechanically ventilated patients, implying that muscle quality as well as muscle quantity are prognostic factors in intensive care units (Looijaard et al., 2016). Low muscle density has also been associated with poor survival and increased risk of hospitalization (Cawthon et al., 2009; Looijaard et al., 2016). Myosteatosis implication in the prognosis and survival post liver transplantation (Hamaguchi et al., 2014), increased fatty infiltration of cardiac muscles observed in diabetes and correlation with diastolic dysfunction have been documented (McGavock et al., 2007).

As first suggested by the Leiden Longevity Study, myosteatosis is a novel marker of impaired survival (Wijsman et al., 2012). This study revealed that offspring of Caucasian nonagenarian siblings predisposed to longevity had lower myosteatosis compared to matched controls. Myosteatosis’ association with greater risk of mortality has been observed in several large studies, even after adjusting for muscle mass. Various have been conducted in the general population (Miljkovic et al., 2015; Reinders et al., 2015; Zhao et al., 2016; Santanasto et al., 2017), while others focused on specific populations such as hospitalized older adults or those with chronic illness (Locke et al., 2017; Perkisas et al., 2017). Not all studies, however, have found this relationship as some have reported no association between mortality and myosteatosis (Cesari et al., 2009; Murea et al., 2018). These conflicting findings may relate to the definitions and measures used to assess myosteatosis, as well as the muscle(s) examined. It is currently unknown why the relationship between myosteatosis and mortality exists, and more mechanistic studies may be needed to clarify these aspects.

**Figure 1:**
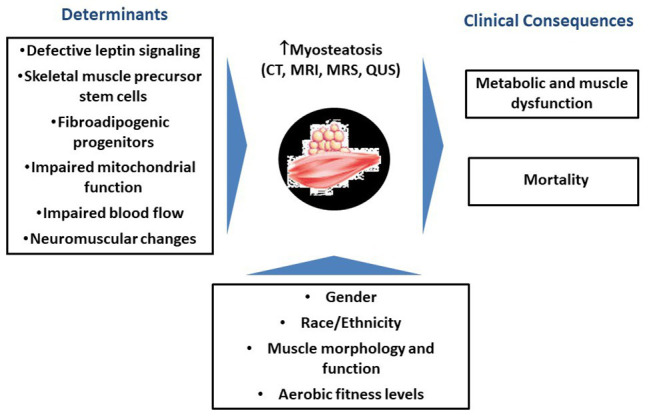
Schematic representation of potential determinants and clinical consequences of increases in myosteatosis with aging.

Figure 1 is a schematic representation of potential determinants and clinical consequences of increases in myosteatosis with aging.

## Standardized Measures of Body Composition and Imaging Approaches to Myosteatosis

### Cutpoints for Assessing Lean Mass, Strength, and Predicting Poor Outcomes

Efforts to create cutpoints for muscle mass and muscle strength from population studies (community-dwelling older adults) recommended cutpoint for grip strength of 26 kg for men vs. 16 kg for women, and for appendicular lean body mass (measured with DXA) of 19.75 kg for men vs. 15.02 kg for women (Alley et al., 2014; Cawthon et al., 2014). Data (thigh cross-sectional area from CT or appendicular lean mass from DXA) from the AGES in Reykjavik and Health ABC studies have been used to explore how adipose infiltration in muscle relates to either sarcopenia or dynapenia (loss of muscle strength; Harris et al., 2007). Across studies, both muscle strength and muscle area were consistently associated with risk of mortality. When muscle attenuation or IMAT was examined, men showed a clear positive association with mortality, while in women only muscle attenuation (not IMAT) was associated with mortality. Similar pattern was noted for mobility-disability, with both muscle mass and strength being protective factors across gender and across studies. The association between myosteatosis and mobility-disability was observed among men only. In the Health ABC study, muscle attenuation was linked to increased risk of mobility-disability in women. Despite consistency of the association with strength and muscle area, the association between muscle attenuation and IMAT was more consistent for death than for risk of mobility-disability. Low muscle mass and poorer strength continued to be independent risk factors. From this analysis, muscle likely had very little effect on the cutpoints for sarcopenia (by DXA) or low strength. It appears that these were operating in slightly different ways and that strength and low mass remained associated with outcomes even when controlling for myosteatosis. As a risk factor for disease, disability, or other health outcomes, myosteatosis is a contributing factor for mobility-disability. Its assessment, however, is traditionally missing from analyses of sarcopenia and dynapenia. Its inclusion in such analyses is needed to further enhance the understanding of the importance of both sarcopenia and dynapenia.

### Quantitative Imaging of Myosteatosis and Implications for Physical Performance

Not all imaging modalities are capable of quantifying myosteatosis by all definitions. Myosteatosis quantification can be performed using invasive analysis such as a muscle biopsy, but it can also be conducted noninvasively using imaging devices such as CT, peripheral quantitative computed tomography (pQCT), and MRI. Quantitative ultrasound (QUS) is emerging as a feasible, inexpensive, and easy device to use for the same purpose. While inter and intramuscular adipose tissue are identifiable with CT and MRI, IMCL requires more specialized imaging techniques to visualization and quantification such as magnetic resonance spectroscopy (MRS) or muscle biopsies. Imaging is desirable for research because it is noninvasive and facilitates visual inspection of fat distribution in and around muscles. However, there is no established gold standard device to quantify myosteatosis. Table 1 summarizes advantages and disadvantages of imaging modalities in assessing myosteatosis.

**Table 1:** Advantages and disadvantages of imaging modalities in assessing myosteatosis.

	Advantages	Disadvantages
Computed tomography (CT)	Differentiates SAT and IMAT Axial and appendicular anatomic sites can be scanned Excellent reproducibility and reliability of muscle and adipose tissue attenuation Allows 3D reconstruction	Cannot directly measure the location of fat storage or lipid droplets within muscle High cost Limited access Ionizing radiation Not portable
Peripheral quantitative computed tomography (pQCT)	Differentiates SAT from intramuscular adipose and IMAT Quantifies muscle density Lower cost Limited ionizing radiation Portable device	Axial and proximal appendicular anatomic sites cannot be scanned Individual muscle groups cannot be segmented Cannot distinguish between intramuscular fat and IMAT
Magnetic resonance imaging (MRI)	Muscle compartments can be segmented Differentiates SAT, intramuscular adipose, and IMAT High quality visualization of IMAT distribution Spectroscopy permits IMCL quantification	Cannot measure muscle density High cost Limited access Not portable Cannot be used in individuals with metal implants Lack of standardized protocols for scan acquisition and adipose tissue quantification
Quantitative ultrasound (QUS)	Reliable measures of muscle thickness and echogenicity Axial and appendicular anatomic sites can be scanned Lower cost No ionizing radiation Portable device	Inter-machine validity unknown Consistency relies on probe placement, pressure, and angle of incidence Cannot distinguish between intramuscular fat and IMAT

CT is one of the most widely used imaging tools to indirectly assess myosteatosis, especially in large cohorts. The CT analysis of myosteatosis is based on a Hounsfield unit, which is a measure associated with the way rays pass through water. Water has a density of zero, higher measurements are denser (i.e., bone), and lower measurements are less dense (i.e., fat). The lower the density, the lower the Hounsfield units and the higher the degree of myosteatosis. While Hounsfield units are not a direct measure of lipid content, the radiation attenuation of muscle shows a high correlation with direct measurements of muscle lipid content by biopsy (Machann et al., 2003; Larson-Meyer et al., 2006). The majority of large studies thus far have utilized attenuation in the thigh muscles due to an ease of analysis and validation of findings with muscle biopsies. However, there is a growing recognition that thigh muscles may not be representative of all muscles in the body and more studies now are moving to other regions including the pelvic girdle and abdomen to assess effects of myosteatosis. CT has excellent reproducibility and reliability that allows 3D reconstruction and selection of axial or appendicular anatomical sites, though the use of a phantom may be necessary to standardize attenuation values across studies. Its major limitation includes the radiation dose to the individual, the cost, and availability. CT is not a portable modality making it difficult to use in some populations and settings. Most importantly, CT cannot directly measure the location of fat storage or lipid droplets within the muscle, for example, intramyocellular vs. extramyocellular, which may be an important distinction for some outcomes.

When used to measure muscles, pQCT devices have the benefit of a lower cost and significantly lower emission of ionizing radiation compared to whole-body CT (Erlandson et al., 2016). pQCT devices are portable, making them useful for research populations in difficult-to-reach locations. While the thigh can be measured in scanners with a larger gantry, the lower leg (generally two-thirds of the length of the lower leg serve as a site for analysis) is most often used with a highest reproducibility in the area where the muscle belly is the largest. Some of its limitations are due to its original design to measure skeletal outcomes rather than soft tissues. Individual muscle groups cannot be isolated with pQCT, and intra vs. IMAT cannot be clearly distinguished. While pQCT permits the assessment of muscle density, there is no standard analysis protocol, and manual segmentation of muscles may be necessary, creating problems with reproducibility and comparison across studies.

**Figure 2:**
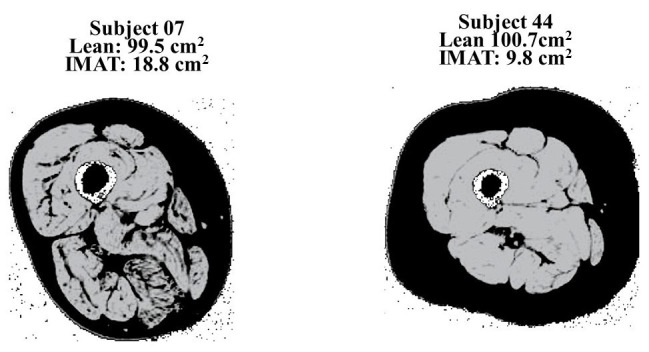
MRI scans that illustrate two women with similar amounts of lean muscle mass, but differing amounts of myosteatosis. Previously published in Addison et al. (2014a).

MRI can be used to quantify myosteatosis with one clear advantage over CT devices: no ionizing radiation. The amount of fat within a muscle can be quantitated (i.e., cross-sectional area of volume), however, at this time a quantitative assessment of muscle density with MRI is not available. MRI provides a high-quality image allowing for muscle groups and fat depots (intra vs. IMAT) to be distinguished and visualized with excellent reproducibility and reliability. While the measurement of IMCL can be done with MRI (utilizing MRS), the device is unlikely to be available at most facilities and cannot be applied to all muscles or in individuals with metal implants such as joint replacements. MRI can also be expensive and time intensive. There is currently a lack of standardized protocols for scan acquisition and software for adipose tissue quantification, making comparison across studies using MRI very difficult. Figure 2 illustrates MRI scans of two women with similar amounts of lean muscle mass, but differing amounts of myosteatosis.

QUS is currently emerging as a low cost and portable modality capable of estimating not only muscle mass but also tissue composition. QUS can provide reliable measures of muscle thickness and echogenicity of a tissue in both the appendicular and axial skeletal muscles (Nijholt et al., 2017). Echogenicity (ultrasound’s brightness) may be used to examine tissue composition (Akima et al., 2016) and is associated with general muscle performance, as well as metabolic parameters (Harris-Love et al., 2018). Increased echogenicity indicates an increase in myosteatosis. The degree of echogenicity also appears to differ based on the bioenergetics of the muscle and its location (Reimers et al., 1993). With aging the magnitude of echogenicity measures are higher in older adults, but the pattern between muscles does not differ between younger and older individuals. When comparing the rectus to the deltoid muscle, the ratio of how bright the rectus muscle is compared to the deltoid is maintained in the 20-year-olds as well as in the 65-year-olds, but the echogenicity absolute values (i.e., change in muscle composition) increase in older adults. This consistent pattern provides an opportunity to identify an easily measured index muscle that can be used to look at changes over time. This would allow for the clinical assessment of myosteatosis and its relationship to muscle performance over time. Myosteatosis quantification may help with earlier determination of those at risk for muscle changes and consequent functional limitations.

Despite many advantages, ultrasound also has limitations. The inter-machine validity as well as the optimal methods and criteria to characterize muscle tissue are currently unknown. There are also issues with the psychomotor components of scanning such as consistency of probe placement, pressure, and the angle of incidence. Tools are currently under development to provide real time feedback to mitigate these challenges. These tools might be scalable in the future and help with implementation of this technology quickly into a variety of clinical settings. Given the current technologies and the lack of a standardized approach to assess myosteatosis, there is a need to focus on identifying best technologies that are reliable, easy to use, cost-effective, applicable in clinical settings. Defining which muscles are the most clinically relevant to evaluate the relationships of myosteatosis to aging and related outcomes, as well as the effectiveness of potential interventions overtime are major areas for further investigation.

Across all measurements of myosteatosis, longitudinal measurement of muscular fat infiltration is particularly challenging. The variety in muscle analysis software and variations in fatty infiltration nomenclature makes comparison across studies difficult. Moreover, inconsistencies in image acquisition protocols across technologies, including muscle groups and regions measured have substantial effects on analysis protocols and results interpretation. There is a need to establish standardized image acquisition and analysis protocols, which would streamline thresholding algorithms, nomenclature, and clarify how to account for visible motion artifact. Standardized image acquisition and analysis would facilitate addressing important questions such as: are trajectories of change in intramuscular and IMAT depots associated with declines in physical function? What is “normal” vs. pathological myosteatosis? Longitudinal studies are needed to understand not only how to develop interventions to reverse myosteatosis but also to ensure that myosteatosis reversal is clinically meaningful for the individual in terms of health outcomes.

### D_3_-Creatine Dilution Method to Determine Muscle Mass

Major methods utilized to evaluate muscle mass include MRI, DXA, and a 24-h urine collection. These can be done on a research basis, but they have limitations when applied to a very large audience based on availability, cost, and in the case of a 24-h urine collection, subject adherence. Creatine is a key component of muscle energetics and acts on the regeneration of adenosine triphosphate (ATP) from adenosine biphosphate (ADP) and has a unique feature of being 95% exclusively located in muscles. Exploratory studies in rats targeted the demonstration of D_3_-creatine excretion in urine as a marker of total body creatine pool size and muscle mass. A strong correlation (*R* = 0.952) between lean mass by quantitative magnetic resonance (QMR) and creatine pool size as calculated by D_3_-creatine enrichment in urine in both single and repeat dosing was shown (Stimpson et al., 2012, 2013).

In single-dose human studies where muscle mass was evaluated in all subjects by MRI (serial cross-sections) and total lean body mass as well as appendicular lean mass were evaluated by DXA, comparison of DXA vs. MRI showed a great deal of bias in that measurements resulted in almost a doubling of the total lean mass estimated by DXA. This was largely due to the inclusion of non-muscle tissue in total lean body mass (soft tissue, intestines, liver, lungs, and body fluids). When looking at just appendicular lean mass estimates with DXA, there was a good correlation (*r* = 0.957), but DXA underestimated lean mass resulting in a significant lower amount of appendicular lean mass than the actual value was per MRI. However, while appendicular lean mass shows a reduction with DXA vs. MRI, the total body scan shows a value that is nearly double that of the MRI muscle mass. Nevertheless, the D_3_-dilution method actually comes relatively close for estimates of muscle mass when compared to the MRI method (Clark et al., 2014).

A repeat dose study in humans was used to assess whether the D_3_-method could be repeated over a 3–4 month interval, to follow-up response of therapeutic use (e.g., drugs), and to observe how older subjects would respond in view of muscle wasting secondary to aging or chronic disease. This study was conducted in subjects housed on the inpatient unit for 5 days. Results were significant and showed: (1) variation of dose excreted in urine by sex. Median loss was 3.5% among men and 25% among women. (2) Majority of excretion occurred in the first 24 h. (3) Median time to isotopic steady state of excreted D_3_-creatine was 26 h in older men and 52 h in older women. (4) Muscle mass estimated using D_3_-creatine excretion demonstrated a strong correlation with MRI. Similarly, DXA total lean body mass showed the near doubling based on the bias alluded before. Intra-subject variability, i.e., variability between their first and second studies, showed a large standard deviation for D_3_-creatine dilution compared with the MRI cross-sections or total lean mass by DXA, thus indicating that D_3_-method may not be appropriate for some pre-post or longitudinal studies (Clark et al., 2018).

The D_3_-creatine method is emerging as a new method to quantify lean tissue with less bias than whole body DXA. The D_3_-creatine method showed that it strongly correlates with MRI estimate of whole-body mass. The method potentially provides a simple approach to assess total body lean tissue with easy access and acceptable accuracy, which could be used in the future to estimate total lean mass in clinical populations. This could be important in populations where the use of DXA to estimate lean mass may mask the true loss of muscle mass. Estimates of lean mass using data from DXA are probably not a good approach to pursue as very large bias could lead to the belief that subjects have more muscle mass than present. It should be noted, however, that when examining the intra-subject variability between repeat measures, there was a large standard deviation for D_3_-creatine dilution compared with the MRI cross-sections or the total lean mass by DXA. So, its use may be hampered by such variability (Clark et al., 2018).

### Shape and Appearance Modeling of Dual-Energy X-Ray Absorptiometry and 3D Optical Scans to Study Lean Mass

DXA is a commonly available option for clinical use due to its lower radiation levels and ease of use. However, while studies have attempted to use DXA to derive muscle density as a measure of myosteatosis, they have thus far been unsuccessful. Using data from the Health ABC study, Malkov et al. (2015) used DXA-derived estimates of mid-thigh muscle attenuation, SAT thickness, and thigh muscle cross-sectional area, with the goal of obtaining attenuation values that would be analogous to those from CT scans. The DXA estimates were compared to CT-derived measures, and the association of both CT and DXA measures with fracture risk was calculated. Although DXA derived subcutaneous fat thickness was a strong marker for hip fracture, thigh muscle attenuation evaluated by DXA had no predictive value for fracture risk.

Despite the inability to accurately determine muscle attenuation and myosteatosis, it is possible to extend DXA’s usefulness beyond just traditional measures of bone or lean mass. It is likely that there are differences in the way that the body is shaped in those with high and low muscle density and unlikely that someone with poor-quality muscle and high levels of myosteatosis physically resembles someone with high-quality muscle. Hinton et al. (2017) utilized DXA from the National Health Nutrition and Examination Survey (NHANES) to generate radar charts that examined the body symmetry of both lean and fat mass. The development of such charts allows for a quick visualization that if instituted clinically could support identification of patterns that arise in conditions such as sarcopenia or myosteatosis. Moreover, these charts could be used to observe changes over time resulting from aging or interventions.

Current work on shape and appearance modeling by DXA and 3D optical scans is focusing on a definition of phenotypes for poor muscle quality. For example, DXA can be used to calculate total body volumes (Wilson et al., 2013), a potentially useful application for examining disease risk associated with certain body phenotypes such as those with a high trunk to leg volume ratios. High trunk to leg volume has a strong association with both diabetes and mortality that is independent of fat distribution even among those with a normal BMI (Wilson et al., 2013). While obesity is associated with an increased risk for cardiovascular disease, there is a need for further stratification of patients’ risks that existing body phenotypes cannot provide (Vecchie et al., 2018). One approach to this is to examine prevalent clusters of total body fat distribution in men and women and their relationship to metabolic health. Using statistical appearance modeling, applied to quantitative DXA scans, and principal component analysis, the associations of body shape and metabolic outcomes were examined to identify individuals at high risk for metabolic disease (Shepherd et al., 2017). Importantly, these detailed models of body shape and tissue distribution with DXAs were able to accurately identify sex and race, as well as predict mortality. Future work with DXA may allow for the development of clinical phenotypes to predict high levels of myosteatosis in some individuals.

## Innovative Relevant Areas of Investigation

### Myosteatosis and Metabolism: Role of Skeletal Muscle Mitochondrial Function

The skeletal muscles and the liver are the major organs involved in the cellular and molecular basis of the insulin resistance process, and the skeletal muscle is a major site of insulin stimulated glucose disposal. Fatty infiltration in muscles is a potential target as a factor affecting glucose transport in those with diabetes, pre-diabetes, or insulin resistance. The prevalence of pre-diabetes and diabetes is over 50% in the United States (Menke et al., 2015). The mechanisms for insulin resistance have been extensively reviewed by Petersen and Shulman (2018); multiple MRS studies have revealed that the rate-controlling step for insulin-stimulated glycogen muscle synthesis in those with diabetes or insulin resistance is glucose transport. IMCL is a strong predictor of insulin resistance in sedentary individuals (Krssak et al., 1999) and muscle insulin resistance can be induced within 5-hours of a lipid infusion in healthy individuals (Dresner et al., 1999), and a fatty acid derived-metabolite (e.g., DAG) has been suggested to be responsible for mediating reduced insulin signaling and insulin action.

These findings have been replicated in animal studies (rodents and rats), further expanding our understanding of the defect involving insulin signaling and action (Griffin et al., 1999; Shulman, 2014; Szendroedi et al., 2014). Infusion of lipid in healthy rats or mice results in profound insulin resistance within 4–5 h, with plasma fatty acids going up. Petersen et al. (2003) matched healthy 70- and 80-year-olds (mean BMI 25 kg/m^2^) with young, healthy, lean individuals to examine differences in insulin action and resistance. In response to the 2-h glucose tolerance test, the 70-year-olds showed slightly higher plasma concentrations of glucose, but much larger increases in the plasma concentration of insulin reflecting whole body insulin resistance. The healthy older adults also had twice the amount of IMCL compared to the younger individuals as assessed by ^1^H MRS, which was associated with a 35–40% reduction of muscle mitochondrial fat oxidation as assessed by ^13^C MRS and muscle mitochondrial ATP synthesis as assessed by ^31^P MRS (Petersen et al., 2003). In order to investigate a potential causal role of reactive oxygen species (ROS) to age-associated reduced mitochondrial function in the pathogenesis of increased IMCL and muscle insulin resistance, mice with catalase targeted to the mitochondria were studied (Lee et al., 2010). Like humans, it was found that aged wild type mice develop muscle insulin resistance, which was associated with increased muscle lipid (TAG/DAG) content (but no change in muscle ceramide content) and reduced muscle mitochondrial function. Interestingly, all these effects of aging were abrogated in mice with targeted overexpression of catalase to the mitochondria. Taken together, these pre-clinical data suggest that mitochondria function decreases with aging, in part due to cumulative ROS damage, and predisposes individuals to ectopic lipid (DAG) build-up in muscle and muscle insulin resistance.

There are also potential genetic reasons where reductions in mitochondrial activity may be associated with ectopic fat. A study by Knowles et al. (2015) identified a single nucleotide polymorphism in N-acetyltransferase-2 (mouse homolog is NAT-1). In mice with a NAT-1 knockout, there is reduced whole body energy expenditure (Camporez et al., 2017) and reduced oxygen consumption in white adipose tissue, brown adipose tissue, and the liver. The NAT-1 knockout mice also display increased ectopic lipid in the liver and muscle, as well as muscle and liver insulin resistance. These data demonstrate the importance of genetic predispositions to reductions in mitochondrial activity, development of ectopic lipid, insulin resistance, and diabetes. Genetic lesions leading to reduced mitochondrial activity can be further studied to identify genes that protect from ectopic lipid and insulin resistance. Future directions and investigations should include reducing mitochondrial-generated ROS, as most ROS inhibition studies have focused on overall cell ROS inhibition and have not looked specifically at mitochondrially produced ROS.

### IMAT: Innocent Bystander or Active Participant in Muscle Insulin Resistance?

IMAT is strongly related to insulin resistance, however, the exact mechanisms explaining this relationship are still unclear. While several studies have examined the relationships of myosteatosis and insulin resistance using imaging technology to quantify IMAT, few studies have isolated IMAT to examine its role in insulin resistance *in vitro*. IMAT may act in a similar manner to VAT to promote insulin resistance. Using condition media generated from SAT, VAT, and IMAT, it was shown that both VAT and IMAT cause insulin resistance *in vitro*, with IMAT showing a similar potency to VAT (Sachs et al., 2019). This suggests that due to its close proximity to the muscle, IMAT could have a strong local influence on muscle metabolism and insulin resistance. Importantly, the VAT and IMAT in this study were taken from two separate populations. SAT and VAT were taken from patients undergoing bariatric surgery with an average BMI of 45 kg/m^2^. The IMAT came from individuals who were mid-BMI range (around 33 kg/m^2^) and most likely more metabolically healthy. Therefore, these findings, while important, probably underestimate IMAT’s potential to induce insulin resistance and warrant further study.

Why increased IMAT results in insulin resistance is currently unknown, however, IMAT inflammation is a possible reason. The expression of macrophage markers and inflammatory cytokines in IMAT was examined in athletes, lean and obese individuals, and those with type 2 diabetes. RNAseq analysis showed higher levels of macrophage infiltration in those with obesity and type 2 diabetes that correlated with insulin resistance in all individuals. Obese individuals with and without type 2 diabetes also had increased inflammatory cytokine gene expression [plasminogen activator inhibitor-1 (PAI-1), monocyte chemoattractant protein 1 (MCP-1), and tumor necrosis factor (TNF)-alpha-induced protein] that was significantly related to insulin sensitivity measured by clamp. Having greater inflammation with increased macrophage invasion seen in individuals with high levels of myosteatosis could be one reason for the greater insulin resistance (Sachs et al., 2019).

Perilipins are proteins surrounding the lipid droplet that impacts triglyceride lipase activity. In individuals spanning the physiologic range of insulin sensitivity, perilipin 5 gene expression suggested that greater IMAT lipolysis was found in individuals with increased insulin resistance (Sachs et al., 2019). Perilipin 5 is among factors that antagonize lipolysis. While it is unknown whether perilipin 5 gene expression correlates to its protein content, less perilipin 5 protein content in insulin resistant individuals would mean less inhibition of lipolysis, greater lipolytic rate, and greater free fatty acid release that could promote local insulin resistance. While perilipins are not the only factor controlling lipolysis, this is at least consistent with the idea that IMAT in insulin-resistant individuals could be releasing free fatty acid (FFA) locally that are driving muscle bioactive lipid accumulation. These gene expression data were corroborated by *in vitro* measures showing significantly greater rates of lipolysis in VAT and IMAT compared to SAT. This is relevant because in many human studies where there is dietary control, no differences in fasting FFA concentration are observed between groups despite differences in muscle lipid content. It is possible that such differences could be explained by IMAT lipolysis influencing local muscle FFA concentration and bioactive lipid content, but these local differences in FFA content are not found after dilution in the systemic circulation.

There are many unanswered questions regarding the etiology of IMAT, including its cellular origins and how it accumulates with age. What makes IMAT accumulate in the first place? Why does it increase with age? Is it always there and accumulation is due to hypertrophy of the tissue? Or are precursor cells such as FAPs and pre-adipocytes differentiating into IMAT? Could muscle satellite cells trans-differentiate into IMAT? Is all IMAT created equal? How does IMAT respond to lifestyle and pharmacologic interventions? Could IMAT accumulation be prevented with early intervention in younger adults? FAPs are a special type of stem cell population thought to be IMAT precursor cells given their existence in muscle and ability to differentiate into adipocytes in response to a sedentary lifestyle, low physical activity, or pathological conditions (e.g., Duchenne muscular dystrophy). In some *in vitro* studies, FAP-derived adipocytes are actually more insulin resistant than other adipocytes, possibly relating increased myosteatosis with insulin resistance and making this a very active area of investigation.

### Muscle Clocks and Homeostasis

There is growing recognition that circadian rhythm changes with aging (Hood and Amir, 2017). When changes occur in homeostasis or circadian biology, rapid and acute metabolic changes also happen (Depner et al., 2018). Similar changes are observed in rodent models and humans. Typically, energy expenditure is higher during the day and much lower during sleep, but once sleep patterns change, there is also a rapid change in the pattern of energy expenditure over the day which affects body composition and insulin sensitivity. Underlying circadian rhythms is a molecular mechanism called the circadian clock. Circadian clocks exist in virtually all cells in the body including skeletal muscles and is a ubiquitous mechanism that functions to temporarily regulate certain metabolic and cell physiologic processes (Harfmann et al., 2015).

Data from mouse gastrocnemius muscles collected every 2 h for 48 h demonstrate four to ten-fold differences in gene expression from peak to trough in messenger RNA expression over the time of day (Zhang et al., 2014). Similarly, in human studies, muscle biopsies over 24 h, find clock gene expression in the vastus lateralis muscle with a pattern similar to the mouse model when considering the active vs. inactive times of the day. Specifically, genes like *Bmal1*/Arntl peak at the end of the active phase and genes like Per2 peaking at the end of the rest phase. These results are consistent with the human data reported from two timepoints by Zambon et al. (2003) and demonstrate that the molecular clock is functional within human muscles (Perrin et al., 2018).

To address the issue of whether the circadian clock in skeletal muscle has any effects on strength, a muscle specific ablation for *Bmal1*, a core clock gene, was used in a mouse model (Dyar et al., 2014; Schroder et al., 2015). In this model, the circadian clock is intact in the central clock, as well as all non-muscle peripheral tissues. What both studies found was that loss of *Bmal1* only in skeletal muscle was enough to induce significant weakness with diminished forelimb grip strength and a loss of specific force (determined as the maximum isometric force normalized to muscle cross sectional area). The decrease in specific force is a clear indication that there are changes in muscle quality as there were no changes in muscle size. Analysis of myofibrillar proteins has detected changes in the expression of some of the large proteins that contribute to the structure of the sarcomere. In addition, prolonged loss of *Bmal1* function was also associated with increased fibrosis within the muscle. These results demonstrate that factors that disrupt the circadian clock in skeletal muscle lead to changes in gene expression with a downstream impact on muscle structure and function.

Disruption of the muscle clock has also been shown to have significant impact on glucose intolerance and insulin resistance with very small changes in fiber types (Dyar et al., 2014; Schroder et al., 2015; Harfmann et al., 2016). In the muscle specific *Bmal* knockout mentioned previously, a significant increase in glucose intolerance was seen in all mouse models of muscle *Bmal1* knockout. Using an inducible muscle specific *Bmal1* knockout mouse, Harfmann et al. (2016), showed that non-fasted blood glucose (measured every 4 h for about 32 h) in the mice was always elevated compared to controls. When investigating glucose transport *ex vivo*, it was clear that there was no increase in glucose uptake in response to insulin. Further, use of 5-aminoimidazole-4-carboxamide-1-D-ribofuranoside (AICAR) to activate adenosine monophosphate (AMP)-activated protein kinase (AMPK) also did not increase glucose uptake indicating that loss of *Bmal1* affects both exercise as well as insulin-induced glucose uptake. Muscles from *Bmal1* knockout mice exhibit reduced glycolytic enzyme activity (e.g., 50% reduction in both hexokinase and phosphofructokinase activity) and a significant reduction in expression of the glucose transporter type 4 (GLUT-4) suggests that the molecular clock regulates muscle mechanisms that impact glucose uptake in muscle (Harfmann et al., 2016). Targeted metabolomics [glycolysis and the tricarboxylic acid (TCA) cycle] examination showed that muscle glycogen was increased, but this is likely due to decreased expression of the rate limiting enzyme for glycogen breakdown (glycogen phosphorylase). Pyruvate dehydrogenase (PDH) activity is also reduced, leading to increased levels of lactate (Dyar et al., 2014). All metabolomics data were consistent with the concept that metabolic flux through glycolysis is decreased. The muscle was still able to maintain ATP levels, but the ADP levels were down, which relates to energy charge. These studies also found that levels of several different amino acids were elevated, suggesting that the muscle may be relying on the breakdown of amino acids to support the TCA cycle needs for energy.

It is now apparent that many factors can contribute to disruptions in circadian clock function in muscle including aging, inflammation, and obesity. Studies are also emerging that link physical activity or exercise to muscle clock function (Wolff and Esser, 2012; Sato et al., 2019). Thus, the more sedentary individuals become the more likely they are to have their muscle clocks affected, which, in combination with the reduced physical activity, may lead to myosteatosis. In addition to exercise, factors such as time of feeding can modify the clock settings in skeletal muscle and other peripheral tissues such as the liver and this occurs independent of light exposure (Paschos and FitzGerald, 2017). These recent findings suggest that lifestyle factors may have a critical role in trying to help clocks stay synchronized throughout the body.

### Muscle-Bone Interaction: Myosteatosis and Bone Marrow Adiposity

Bone marrow is another tissue where fatty infiltration occurs, and this adiposity shares many similarities with myosteatosis. Normal human bone marrow is about 10% of all fat stored and 30% of the marrow volume in young individuals. Similar to muscle, adiposity in the bone marrow occurs with aging and up to 70% of marrow in older adults may be adipocytes (Fazeli et al., 2013). This change begins around the age of 13, which is the time of peak bone acquisition.

Unlike muscle, there are no ectopic lipid droplets in the marrow tissue itself; rather, lineage tracing indicates that bone marrow adiposity is made of adipocytes that differ in size and in location. However, lipid droplet formation may exist inside osteoblasts, similar to IMCL accumulation. Comparable to the impact of the location of muscle lipid depots on metabolism and muscle function, emerging evidence indicates that the distribution of marrow adipocytes may also have differential effects on the skeleton. There is also genetic programming of increases in bone marrow adipose tissue. The mass and strength of both muscle and bone decline with aging and is accompanied by an accumulation of adipose tissue. Evidence suggests that many of the factors observed to stimulate bone marrow adipogenesis (disuse atrophy, estrogen deficiency, leptin deficiency, and glucocorticoid treatment) also induce myosteatosis (Hamrick et al., 2016; Veldhuis-Vlug and Rosen, 2018). As in myosteatosis, the stem cell for the bone marrow adipocyte is not known. Investigations of bone marrow adiposity could provide a critical clue to what is occurring in muscle.

In the aging mouse, the marrow cavity is almost fully replaced by adipocytes, clearly recapitulating findings with the aging phenotype in humans. In the C3H mouse, early progression of bone marrow adipose tissue occurs (Scheller et al., 2015). While this may initially appear negative, the C3H mouse has not only a tremendous increase in marrow fat, but a much denser skeleton with higher bone mass. That increased marrow adiposity may have potential benefits for some outcomes but also suggests there may be some unexamined positive benefits of myosteatosis that require further study. Experimental findings on reducing bone marrow adiposity may inform mechanisms and interventions for reducing myosteatosis.

## Lifestyle and Therapeutic Interventions

### Potential Preventative and Therapeutic Approaches

For a long time, research focused on increasing muscle mass, but a more important issue to be addressed seems to be the change in muscle quality. Even physically active healthy older adults demonstrate some amount of fat infiltration. It is not currently possible to revert completely the effects of aging. The LIFE pilot study suggests that even an attenuation of increases may be clinically important as it appears that increased myosteatosis is a phenotype of aging that may be prevented with modest physical activity. Although physical activity looks like a promising approach to reducing IMAT, more studies are needed in large controlled trials. The mechanisms of preventing or potentially reversing muscle fat accumulation, whether through metabolism and reduction of inflammation, or other methods, have not yet been definitely established. Most studies have thus far focused on muscles in the thigh and lower extremities, but fat infiltration occurs in multiple localizations, making investigation of other muscles important.

In a sheep model of rotator cuff injury, the pennation angle of the muscle fibers changes when there is atrophy. This creates a space within the fiber, and this space is filled with fat (Meyer et al., 2004). When muscle fibers are mechanically stimulated, the accumulation of fat is reduced. The mechanical stimulation by itself seems to be counteracting the accumulation of fat (Rubin et al., 2007). However, one potential consequence of increased myosteatosis is likely a decreased ability to activate a muscle, making it more difficult to stop the accumulation of fat once it has started. At least one study has demonstrated that when fatty infiltration increases, there is a decrease in the central activation ratio of a muscle, meaning a diminished ability to fully voluntarily activate a muscle (Yoshida et al., 2012). This may impact the ability to reduce IMAT with physical activity. Among older adults participating in an intensive eccentric resistance training program, only those who had low levels of myosteatosis at the start of the intervention made changes in their muscle quality. This suggests that older adults with high levels of myosteatosis may be impaired with making such changes (Marcus et al., 2013). Alternative approaches used in conjunction with exercise may be needed for some older individuals to improve muscle quality.

A vibration platform may be useful for older adults with limited ability to fully voluntarily recruit their muscles due to illness or increased myosteatosis. When vibration is applied to the feet, it stretches the muscle, and the response of the muscle is several repeated contractions. Electromyography confirms muscle activation induced by vibration. Some studies show that vibration is even more effective than resistance training (Verschueren et al., 2004) at improving dynamic strength with an isokinetic dynamometer. While logically it could be assumed that vibration may have some effect on myosteatosis, this has not yet been examined and may be a promising area for future research.

The use of pharmaceutical intervention combined with lifestyle intervention to delay or reverse myosteatosis is still largely unexplored. Treatment with an antimyostatin antibody may potentially be one approach. In mice, even short-term treatment (approximately 4-weeks) with an antimyostatin antibody results in increased whole body muscle mass, grip strength, and insulin action (Camporez et al., 2016). Triglycerides and ectopic lipids also decrease with increased sensitivity in both muscle and liver. The optimizing body composition for functioning in older adults (OPTIMA) study (Shea et al., 2011) attempted to engineer weight loss to optimize its functional benefits in older adults by using pioglitazone to accelerate the loss of VAT. In this large pilot study of 48 men and 40 women, 65–79 years of age, with an indication for weight loss, all got a weight loss intervention, but half received the drug pioglitazone and half a placebo. Individuals were also randomized to resistance training or no training, to maximize muscle retention during weight loss and to see whether that was likely beneficial. After the 4-month intervention, changes in fat and lean mass, the size of ectopic fat depots, and functional measurements were examined. Over the course of the intervention an average of 6.5% total weight loss was achieved, with approximately 14 and 9.7% total body fat loss in men and women, respectively. Intramuscular fat in the thigh decreased in proportion to overall fat loss, but no significant change in muscle attenuation was seen. When comparing pioglitazone/no-pioglitazone in men, the use of pioglitazone doubled the amount of VAT loss in men, but in women, no statistically significant difference in VAT loss was detected. There was not a strong benefit or harm from pioglitazone with regards to myosteatosis with no statically significant difference in changes with and without the drug. These results indicate that in men, the use of pioglitazone engineered weight loss to theoretically have superior benefits. Pharmaceutical and lifestyle combination interventions may be beneficial in examining myosteatosis, however, a sex specific approach may be necessary. In addition, more human studies are needed to investigate pharmaceutical approaches.

### Calorie Restriction

Cross-sectionally, adiposity and obesity-related measures are more strongly related to mobility in community dwelling older adults than measures of muscle mass (Kritchevsky, 2014). With the exception of the SAT compartment, fat accumulation appears deleterious. Intramuscular fat or attenuation in the legs is associated with decreased strength, increased insulin resistance, and increased disability risk. Pericardial fat is related to increased coronary calcification and stiffness in large arteries (McClain et al., 2013). Fat in the area of the renal sinus is associated with harder-to-control blood pressure (Chughtai et al., 2010). VAT associations are also well-known for their links with inflammation, insulin resistance, and functional impairments. Fatty liver is related to increased inflammation and glucose deregulation. Choi (2016) found that obese adults had larger fiber cross-sectional area. It appears that in obese individuals, there is a direct impairment of force generation in individual muscle fibers, though the cause of this is still unknown. IMAT in older persons can also be a source of senescent cells; as reported previously, the frequency of P16-expressing cells in IMAT was strongly and inversely associated with functional measures like gait speed and SPPB score (Justice et al., 2018).

The fat depot specific effects on function can also be seen in the context of trials of calorie restriction. Five to six months of moderate calorie restriction leads to about a 7–8% loss of total mass, with associated changes in VAT, thigh IMAT, and pericardial fat (Brinkley et al., 2011). However, there is variability between participants in depot-specific changes. Change in VO_2_max is strongly associated with decreases in the pericardial fat depot, again suggesting a specificity in where fat is lost and the related functional benefits. The number of seconds required to complete five chair-rises is also strongly associated with changes in intramuscular adipose tissue. For the 4-meter walk, both VAT and IMAT loss are linked to improved walk speed, but this relationship did not significantly affect pericardial fat (Brinkley et al., 2011).

How fat depots relate within individuals is another potential area of investigation. With weight loss all fat depots are affected, but some people lose more fat from some depots than from others. In a 4-month, calorie restriction-induced weight loss program obese individuals lost about 10% of their body weight (Shea et al., 2011). While they experienced a loss of muscle mass, their muscle composition also changed, with their normal-density muscles relatively preserved, but the low-density component (indicting increased myosteatosis) lost during the program. These findings suggest that calorie restriction may be one way to reduce fatty infiltration in the muscle. Of note, several weight loss studies in older adults show that despite lean tissue loss, strength is maintained, and this may be at least partially attributable to a loss of low-density muscle tissue with weight loss.

Considerable differences exist between adipose tissue depots and their relationship to metabolic dysfunction. The same seems true for different IMAT depots, such as differences between muscle groups and location within the muscle. High degrees of myosteatosis in the large locomotor muscles of the lower extremities may have a different role or influence on whole body metabolism compared to smaller muscles in the forearm. Once it is clearly established why and how IMAT develops, targeted interventions could be possible to prevent it or attenuate its increase/effects on insulin sensitivity and sarcopenia. Few studies, mostly in older people, demonstrate that exercise can help prevent the accumulation of IMAT; however, less work has been done in younger individuals or in utilizing pharmaceutical interventions.

## Symposium Summary

The role of myosteatosis in aging muscles and metabolic disease is complex and associated with a multitude of negative clinical outcomes. Discussions carried out during the interdisciplinary workshop is summarized as follows. There is a need for a standard definition of myosteatosis that can be applied across studies and populations. Standardized tools and measurements including new imaging techniques that can be easily used are necessary to enhance the clinical applicability of findings from studies of myosteatosis. Longer longitudinal cohorts are necessary to examine a variety of muscles across multiple racial, ethnic, and disease populations. Identification of which muscles should be examined and measured (how), as well as the establishment of widely accepted cutpoints will enhance clinical practice toward identification of individuals at risk for poor outcomes. Multidisciplinary collaborations that examine the combination of diet, physical activity, and medications are essential to manage individuals at risk. These interventions are often examined as separate interventions but bringing them together will likely result in more useful information and provide new treatment options. Further investigation into the commonality between myosteatosis and changes that occur in other cells in the body may also lead to new discoveries and treatments options. Studying myosteatosis role as a newly defined independent risk factor should be expanded. Anatomic and physiologic studies relating the measures discussed in this report to bench measures are necessary. Bridging molecular, pathologic, and population studies can help identify what is being examined in the tissue and promote a better understanding of the risk factors and mechanisms for myosteatosis, whether the condition is reversible and to what extent. Opportunistic opportunities like cancer populations, shoulder injury patients, bariatric surgery patients, and conditions that may accelerate myosteatosis would considerably expand our knowledge and open an array of research prospects in the field. Best practices for the method of acquisition for both cross-sectional and longitudinal studies, having to do with the acquisition of the data, the analysis of the data using the same cutpoints nomenclature, and then a cross-talk between the imaging modalities would, for example, ensure that what is being assessed from CTs is the same being assessed with MRIs. In conclusion, the workshop identified many research gaps, new questions and opportunities and provided new insights for the identification, causes, consequences, and potential preventive and therapeutic approaches for myosteatosis.

## Author’s Note

The participation of this individual or the materials should not be interpreted as representing the official viewpoint of the U.S. Department of Health and Human Services, the National Institutes of Health, or the National Institute on Aging, except where noted.

## Author Contributions

RC-d-A conceptualized and organized the workshop. RC-d-A and OA drafted initial version of this manuscript. All authors contributed to the article and approved the submitted version.

### Interdisciplinary Workshop Expert Panel Members

Odessa Addison, Department of Veterans Affairs and University of Maryland School of Medicine

Bryan Bergman, University of Colorado

Richard V. Clark, US Anti-Doping Agency

Joanne Elena, National Cancer Institute

Karyn Esser, University of Florida

Luigi Ferrucci, National Institute on Aging

Bret Goodpaster, Translational Research Institute for Metabolism and Diabetes

Tamara Harris, formerly with the National Institute on Aging,

Michael Harris-Love, Department of Veterans Affairs and the University of Colorado

Steve Kritchevsky, Sticht Center for Healthy Aging and Alzheimer’s Prevention Wake Forest School of Medicine

Amanda Lorbergs, Canadian Frailty Network

Iva Miljkovic, University of Pittsburgh

John Shepherd, University of Hawaii Cancer Center

Gerald Shulman, Yale University School of Medicine

Clifford Rosen, Maine Medical Center Research Institute

## Conflict of Interest

The authors declare that the research was conducted in the absence of any commercial or financial relationships that could be construed as a potential conflict of interest.
